# Editorial on the Special Issue “Chitosan Functional Hydrogels: Synthesis and Applications”

**DOI:** 10.3390/gels9070524

**Published:** 2023-06-27

**Authors:** Gibson S. Nyanhongo

**Affiliations:** 1 Department of Agrobiotechnology, University of Natural Resources and Life Sciences (BOKU), 3430 Tulln an der Donau, Austria; g.nyanhongo@boku.ac.at; 2 Department of Biotechnology and Food Technology, Faculty of Science, University of Johannesburg, Johannesburg 2092, South Africa

Chitin, a polysaccharide composed of β-(1–4)-linked 2-deoxy-2-acetamido-d-glucose units, is found in cell walls of different organisms, including crustaceans, fungi, insects, some algae, microorganisms, and some invertebrate animals, and its deacetylation into chitosan confers it with incredible chemical versatility allowing it to be processed into numerous products. This has expanded the applications of chitosan in the agriculture, food, healthcare, pharmaceutical, medicine, environmental, and wastewater treatment industries, as well as in many industrial special functional polymers and materials such as adhesives, auxiliaries for dyeing, and paper making. One of the most prominent, well-established, and quickly expanding fields of application is the biomedical application of chitosan-based hydrogels in producing homeostatic agents for wound management and products promoting wound healing.

To aid readers, Pellis et al. [1] summarize chitosan’s attractive properties, such as biodegradability, biocompatibility, non-antigenicity, bioadhesion, hemostatic activity, and its ability to protect wounds from microbial infection, maintain a moderately moist healing environment, absorb wound exudate, and improve the local microenvironment of the wound, promoting tissue regeneration and general wound healing. The authors further provide an overview of (1) different sources of chitin, (2) advances in techniques used to extract chitin and convert it into chitosan, (3) the importance of the inherent characteristics of the chitosan from different sources that makes them suitable for specific applications, and (4) different techniques used to tailor it for specific applications, especially as delivery systems in the medical field. In the context of widening and/or improving the performance of chitosan in biomedical applications, the authors also provide interesting studies which demonstrate the versatility of this polymer in drug delivery, localizing drugs, and increasing drug concentration at the site of action to consequently reduce off-targeted side effects. The important discoveries reported include exploiting the degree of acetylation (DA), degree of deacetylation (DDA), molecular weight (Mw), tailored modification of the reactive amino and hydroxyl groups which influences its physicochemical and biological properties, acid-base behavior, biodegradability, solubility, and reactivity, among many other properties that determine processability and suitability for specific applications. Furthermore, Miron et al. [2] describe a new method that produces calcium carbonate-enriched chitosan from shrimp shell waste, which proposes maintaining the native minerals in the structure of chitin in order to improve the thermal stability and processability of chitosan. These studies show how this impressive list of characteristics of chitosan continuously offers new opportunities for expanding its applications in many areas never imagined before. Although traditionally, chitosan has largely been obtained from food processing waste, the authors also present new sources of chitosan, like as an emerging side-stream product from the breeding of cocoons from the silk industry and as a by-product of protein extraction from insects for food/animal feed industries and fungal fermentation.

The main highlights of this Special Issue are the following studies: one investigating the synthesis of nature-based curcumin-loaded chitosan–PVA hydrogels for conferring antimicrobial properties and promoting wound healing by Chopra et al. [3], the in situ synthesis of *Syzygium aromaticum* essential oil loaded chitosan hydrogels [4], chitosan–gelatin thermosensitive hydrogels for effective delivery of 5FU [5], and the development of chitosan-based vaginal carriers of progesterone [6], chitosan-grafted poly (ethylene glycol) methyl ether acrylate hydrogels for drug delivery [7], and 3-D gallic acid loaded chitosan [8]. For example, interesting findings show that curcumin-impregnated chitosan hydrogels were effective against both Gram-positive and Gram-negative microorganisms and had good binding energy scores and increased interaction with key residues of inflammatory proteins that help in wound healing activity. Similarly, a responsive chitosan-grafted polymer (N-isopropylacrylamide) was formulated in various compositions with polyvinyl alcohol (PVA) as an external crosslinking agent to obtain pH- and temperature-dependent hydrogels. It was shown to withstand shear forces encountered in the vagina due to its mechanism of swelling once in contact with vaginal fluids. Crosslinking mechanisms were thoroughly investigated during the synthesis of chitosan-grafted poly (ethylene glycol) methyl ether acrylate hydrogels for drug delivery [7]. Using ultrasound-assisted emulsification proved to be an efficient one-step to load *Syzygium aromaticum* essential oil into chitosan matrix hydrogels [4]. The produced hydrogels acted positively as gelation-inducing agents, acting as an alternative to the classical chemical crosslinkers to ensure the good physical and chemical stabilization of chitosan. In addition, simple adsorption of gallic acid on three-dimensional chitosan structures conferred antioxidant activity, the mechanical property of 3D chitosan–GA complexes, and a significantly increased antimicrobial capacity [8]. The synthesis of chitosan modified with hyaluronic acid biodegradable polysaccharide hydrogel matrices for cytostatic delivery improved the therapeutic results of patients by prolonging the action of the drug, reducing its toxicity, and providing additional biological activity by polysaccharides [9]. The produced hydrogels had elastic properties that allowed their application in implants. Hydrogel formulations containing cytostatic prepared from N-succinyl chitosan had the highest molecular weight, and the loading of mitomycin C was found to be the most promising for medical application due to their rheological properties and prolonged drug release. This Special Issue also provides a study on the synthesis and application of thermosensitive chitosan-containing hydrogels for veterinary use [10]. The application of this formulation for treating mixed bacterial infections in cows demonstrated the possibility of the in situ formation of a viscoelastic gel and revealed its high therapeutic effect that could be considered for independent veterinary drugs and pharmaceuticals. In another fascinating study, Naik et al. [11] developed a method for producing chitosan-capped silver nanoparticle incorporated Poly(vinyl alcohol) (PVA) hybrid membranes prepared with a solution-casting technique for ethanol dehydration via pervaporation. These hybrid membranes can be efficiently used to separate water from azeotropic aqueous ethanol and can be employed in many applications.

Finally, in order to demonstrate the limitless applications of chitosan in the medical field, a summary of chitosan as a functional biomaterial for designing delivery systems for cardiac therapy has been provided [12]. Cardiovascular diseases are a leading cause of mortality across the globe, and transplant surgeries are not always successful since it is not always possible to replace most of the damaged heart tissues, for example, in myocardial infarction. This review outlined the latest advances in cardiac tissue engineering mediated by chitosan overcoming the barriers in cardiac diseases. The authors reviewed in vitro and in vivo data on drug delivery systems, scaffolds, or carriers fabricated using chitosan for stem cell therapy essential in cardiac tissue engineering. This comprehensive review also summarizes the properties of chitosan as a biomaterial substrate having sufficient mechanical stability that can stimulate the native collagen fibril structure for differentiating pluripotent stem cells and mesenchymal stem cells into cardiomyocytes for cardiac tissue engineering. Cardiac tissue engineering aims to support, replace, or repair cardiac tissue to improve functionality. The major issue with the viability of the implanted cells is addressed by the application of the polysaccharides, of which chitosan plays a major role. Chitosan helps provide mechanical support, avoids the spread of pro-inflammatory agents, and encloses the bioactive materials helpful for the regeneration of the cardiac tissue.

The studies in this Special Issue show the great potential of chitosan in biomedical applications. Exciting solutions that benefit medicine can be foreseen.
